# Conservative Treatment of a Patient with Epidermolysis Bullosa Presenting as Bart Syndrome: A Case Report

**DOI:** 10.1155/2010/302345

**Published:** 2010-05-16

**Authors:** Samet Vasfi Kuvat, Mehmet Bozkurt

**Affiliations:** ^1^Department of Plastic and Reconstructive Surgery, Dicle Medical Faculty, Diyarbakir, Turkey; ^2^Department of Plastic and Reconstructive Surgery, İstanbul Training and Research Hospital, İstanbul, Turkey

## Abstract

We presented a case of a newborn male with aplasia cutis congenita on the lower limb. The case was treated with conservative method. As for the conservative treatment, daily hydrodebridement with 1/200 diluted povidone-iodine and serum physiologic was performed, followed by closure of the wound with a dexpanthenol + chlorhexidine + fusidic acid-impregnated sterile gauze bandage. the followup that occured after three weeks, the wound was completely epithelialized, but a hypopigmented scar remained in the limb.

## 1. Introduction

Aplasia cutis congenita (ACC) is a rare anomaly characterized by congenital absence of the skin, seen in 1 to 2 per 10,000 births [1, 2]. While its etiology is not clear, genetic factors, intrauterine arterial malformation-infection, placental infections, adhesion of amniotic membrane to fetal skin, teratogens (methimazole, misoprostol, valproic acid, benzodiazepine, heparin), fetus papyraceous, and intrauterine pressure or trauma have been argued as the possible causes [2–4]. It can be associated with ACC Adams-Oliver syndrome (additional limb anomalies, cutis marmorata), SCALP syndrome (nevus cebaceus, CNS malformations, aplasia cutis congenita, limbal dermoid, pigmented nevus), Opitz syndrome, and chromosomal diseases [2]. Bart syndrome is the term used to describe the combination of ACC, skin or mucous membranes blistering, and nail anomalies [5]. 

 In this paper, the conservative treatment of a case with Bart syndrome, a rare variant of ACC, is presented.

## 2. Case Report

**Figure 1:**
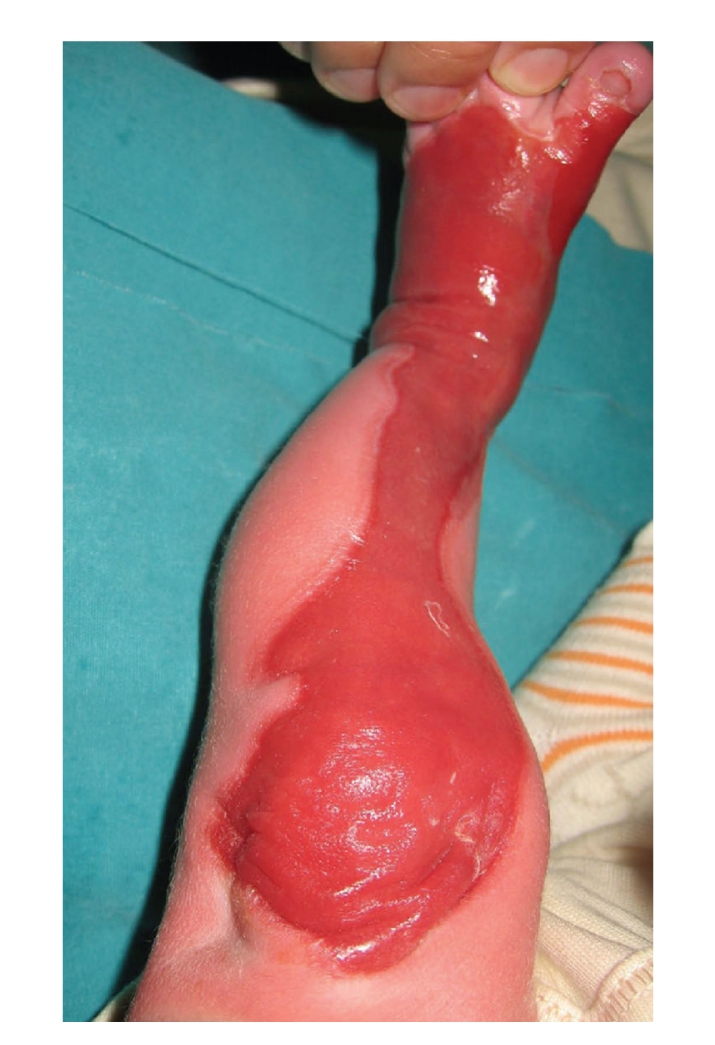
Large skin defects on left extremity at birth.

**Figure 2:**
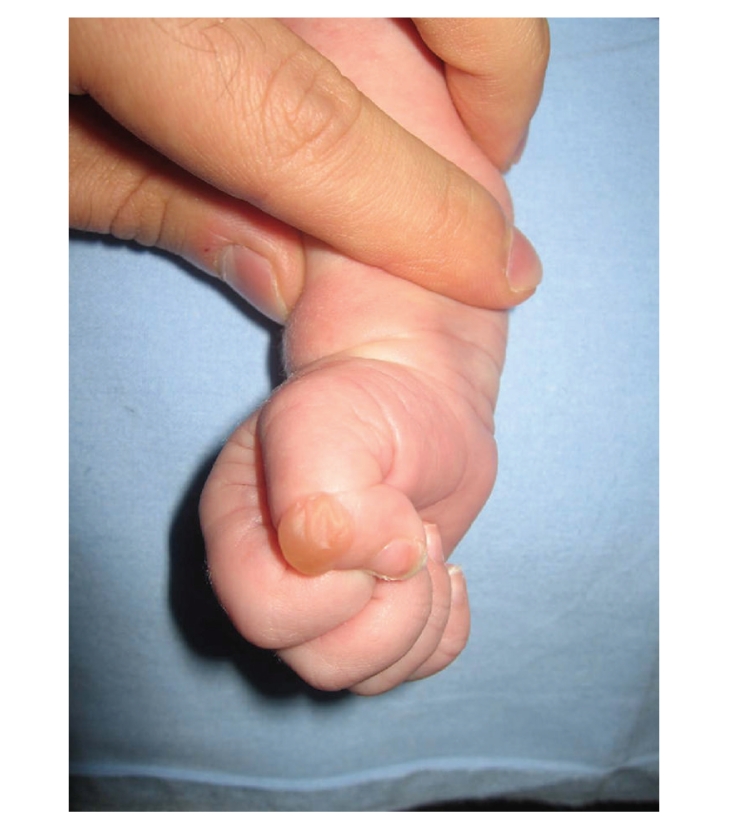
Blistering of the skin on the hand.

A male baby, born vaginally in the 40th gestational age of his mother's first pregnancy, was consulted to our clinic due to anomaly in the left lower extremity. On physical examination of the newborn, sharp edged ACC was observed covering approximately 17 × 8 cm area beginning from the left anterior thigh extending to the distal foot (Figure 1). The defect was covered with an ultrathin translucent membrane. Vascular structures were easily visualized over the membrane. Interestingly, translucency of the lesion decreased in hours, and disappeared at the third day. Some nails of the fingers and the toes were rudimentary. Superficial lesions measuring 0.5 × 1 cm with some deepithelialized areas were observed in the intraoral mucosa, which disappeared at the postpartum first week. Bullous lesions of the skin measuring 0.5 × 1 cm were observed on both hands during the controls, and they disappeared within 7 to 10 days (Figure 2). There was no additional systemic pathology.

There was no maternal drug usage or alcohol-nicotine intake during the gestation, and there was no history of infection or parental consanguinity. However, the father of the patient was learned to have chronic epidermolysis bullosa. The physical examination of the father revealed hypopigmentation and scar formations with some desquamation associated with epidermolysis bullosa in all extremities, especially in fingers and toes, and deformation in fingernails.

**Figure 3:**
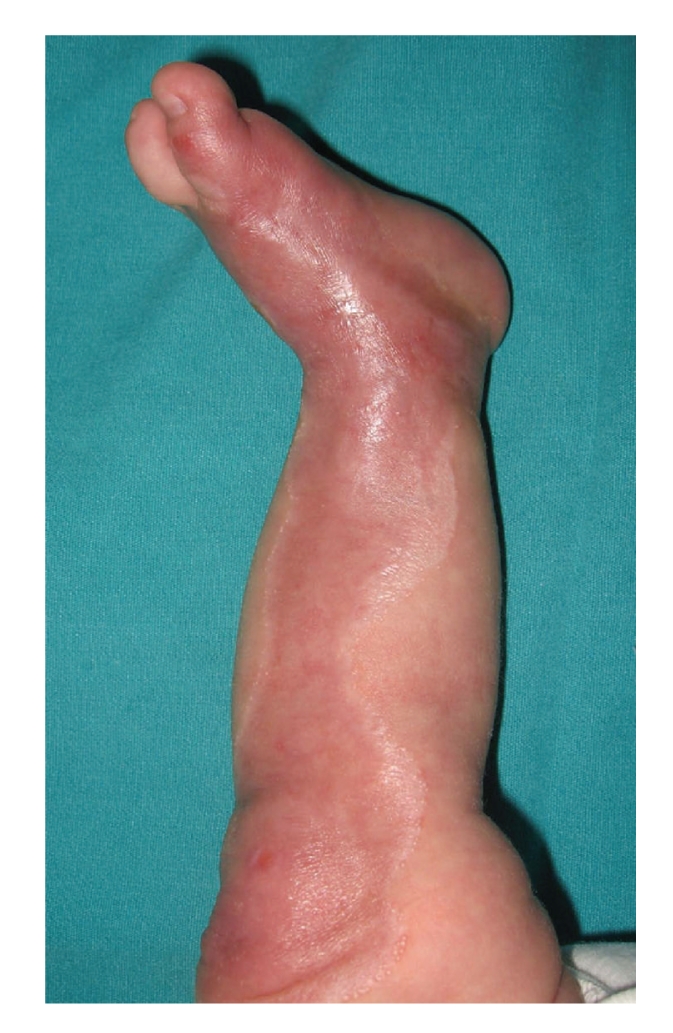
The view of the scars at the end of three-weeks (notice the milia formation).

Following daily hydrodebridement with 1/200 diluted povidone-iodine (100 cc povidone-iodine/20 liters of boiled water) and serum physiologic, the wound was closed with dexpanthenol + chlorhexidine-impregnated sterile gauze bandage. At the end of the first week, culture was obtained upon observation of minimal ulceration on the foot. Fusidic acid was added to the daily treatment upon growth of ampicilline-sensitive coagulase-negative S. aureus in culture-antibiogram. Since there were no systemic infection findings (leukocytosis and fever), systemic antibiotherapy was not performed. After three-weeks, the wound was completely epithelialized, but an hypopigmented scar remained in the area. In addition, the milia was seen in the skin (Figure 3).

## 3. Discussion

Aplasia cutis congenital is an uncommon disorder, characterized by the localized absence of the skin. ACC involves the scalp at a ratio of 85%. Whereas in 15% non-scalp area is affected. Non-scalp aplasia is generally bilateral and symmetrical, and may show familial incidence [2]. Frieden classified ACC into nine groups according to localization, associated anomalies or syndromes, and the inheritance. In our case with familial transition history, coexisting nail deformation, oral deepithelialization, and blisters on hands show that the case should be a typical Bart syndrome [6]. Bart is a type VI aplasia syndrome according to Frieden classification, that exhibits transition in autosomal dominant [7]. We observed no scalp-ACC (Frieden classification type I-II-III-VIII), no associated malformations or syndromes (type IV-IX), no associated with fetus papyraceus or placental infarcts (type V), no caused by specific teratogens (type VIII). The arising formation of blistering was eliminated to choice of type VII. Nevertheless, based on the findings of the recent publications, Bart syndrome should be considered as a clinical variant of dominantly inherited dystrophic epidermolysis bullosa [8]. Further investigations may be focused on determining certainly allocation of related clinical entities.

Considering the possible complications, ACC is an anomaly that should be managed with multidisciplinary approach by the pediatrician, the neurosurgeon, and the plastic surgeon [2]. The most important complications of ACC are infection, hemorrhage, meningitis in the lesions in the vertex and bleeding from the sagittal sinus, thermoregulation and fluid balance disorder in large defects [1]. No complication was observed in our case except minimal ulceration on the dorsal foot.

Surgery is advised in defects larger than 1-2 cm in the scalp which is commonly involved in ACC [3, 9]. Local-regional flap or autograft/allograft applications are preferred methods of intervention. Nevertheless, non-scalp aplasia can be treated with controlled systemic antibiotherapy and conservative management as well [1]. However, scalp/non-scalp aplasia cases which are neglected by the family in the early period may turn into extensive ulcerative lesions until being brought to the clinic [10]. Systemic antibiotherapy is necessary in these cases which may result in death. Since we did not detect systemic infection findings in our case, we did not perform systemic antibiotherapy. In order not to cause neonatal transient hypothyroidism associated with povidone-iodine [11] iodine was diluted 1/200. Despite approaches involving 3 to 4-week hospitalization periods, our case was discharged at the second postpartum day and was followed every three days. Daily wound dressing was performed by the family according to the given instructions.

None of the studies discuss the cause of rapid epithelialization, and therefore it is not clear. It remains unclear since the knowledge about ACC is based on limited number of case reports. We think that one of the causes of rapid recovery may be that the translucent membrane whose histopathology is nonspecific [2] acts like an ultrathin skin graft. For this reason, we can say that nonsurgical methods like repeat AlloDerm grafting or application of cultured keratinocytes [1, 12, 13] is not required in many cases. The recovery time in our case is not different from previous reports involving these methods.

 In conclusion, in cases with non-scalp aplasia that can manifest itself with different clinical presentations, spontaneous recovery with controlled conservative methods seems to be one step ahead of autograft/allograft applications.

## References

[1] Sadowska-Krawczenko I, Korbal P, Piesiewicz A (2004). Aplasia cutis congenita in an infant of the initially twin gestation: a case report. *Medical Science Monitor*.

[2] Shirvany TE, Zahedpasha Y, Lookzadeh M (2009). Aplasia cutis congenita: a case report. *Iranian Journal of Pediatrics*.

[3] Bigliardi PL, Braschler C, Kuhn P, Sigrist J, Buechner S, Rufli T (2004). Unilateral aplasia cutis congenita on the leg. *Pediatric Dermatology*.

[4] Iwayama H, Hosono H, Yamamoto H, Oshiro M, Ueda N (2007). Aplasia cutis congenita with skull defect in a monozygotic twin after exposure to methimazole in utero. *Birth Defects Research Part A*.

[5] Duran-McKinster C, Rivera-Franco A, Tamayo L, Orozco-Covarrubias MDLL, Ruiz-Maldonado R (2000). Bart syndrome: the congenital localized absence of skin may follow the lines of Blaschko. Report of six cases. *Pediatric Dermatology*.

[6] Bart BJ, Gorlin RJ, Anderson VE, Lynch FW (1966). Congenital localized absence of skin and associated abnormalities resembling epidermolysis bullosa. A new syndrome. *Archives of Dermatology*.

[7] Frieden IJ (1986). Aplasia cutis congenita: a clinical review and proposal for classification. *Journal of the American Academy of Dermatology*.

[8] Medenica L, Lens M (2008). Recessive dystrophic epidermolysis bullosa: presentation of two forms. *Dermatology Online Journal*.

[9] Beekmans SJ, Wiebe MJ (2001). Surgical treatment of aplasia cutis in the Adams-Oliver syndrome. *Journal of Craniofacial Surgery*.

[10] Shende R, Khedker MY (1993). Bart syndrome. *Indian Journal of Dermatology, Venereology and Leprology*.

[11] Khashu M, Chessex P, Chanoine J-P (2005). Iodine overload and severe hypothyroidism in a premature neonate. *Journal of Pediatric Surgery*.

[12] Simman R (2004). Letter to the Editor: Management of aplasia cutis congenital non-scalp location. *British Journal of Plastic Surgery*.

[13] Ahcan U, Janezic T, Derganc M (2004). Reply to Letter to the Editor: Management of aplasia cutis congenita in a non-scalp location. *British Journal of Plastic Surgery*.

